# Investigating the impact of socioeconomic status on amyotrophic lateral sclerosis

**DOI:** 10.1080/21678421.2024.2384992

**Published:** 2024-09-01

**Authors:** Ali Shojaie, Ahmad Al Khleifat, Sarah Garrahy, Haniah Habash-Bailey, Rachel Thomson, Sarah Opie-Martin, Sara Javidnia, P. Nigel Leigh, Ammar Al-Chalabi

**Affiliations:** 1 Maurice Wohl Clinical Neuroscience Institute, Institute of Psychiatry, Psychology and Neuroscience, King’s College London, London, UK; 2 Clinical Research Unit, Royal Sussex County Hospital, University Hospitals Sussex NHS, Foundation Trust, Brighton, UK; 3 Department of Neuroscience, Trafford Centre for Biomedical Research, Brighton and Sussex Medical School, Falmer, UK, and; 4 Department of Psychiatry, University of Oxford, Oxford, UK

**Keywords:** Amyotrophic lateral sclerosis, non-motor symptoms, socioeconomic status, Mendelian randomization

## Abstract

Amyotrophic lateral sclerosis (ALS) is a neurodegenerative disease characterized by the gradual death of motor neurons in the brain and spinal cord, leading to fatal paralysis. Socioeconomic status (SES) is a measure of an individual’s shared economic and social status, which has been shown to have an association with health outcomes. Understanding the impact of SES on health conditions is crucial, as it can influence and be influenced by health-related variables. The role of socioeconomic status in influencing the risk and progression of ALS has not been established, and understanding the various factors that impact ALS is important in developing strategies for treatment and prevention. To investigate this relationship, we recruited 413 participants with definite, probable, or possible ALS according to the El Escorial criteria, from three tertiary centers in London, Sheffield, and Birmingham. Logistic regression was used to examine the association between case-control status, socioeconomic criteria, and ALS risk. Linear regression was used to examine the association between age of onset and socioeconomic variables. Two sensitivity analyses were performed, one using an alternative occupational classifier, and the other using Mendelian Randomization analysis to examine association. There was no significant relationship between any variables and ALS risk. We found an inverse relationship between mean lifetime salary and age of ALS onset (Beta = −0.157, *p* = 0.011), but no effect of education or occupation on the age of onset. The finding was confirmed in both sensitivity analyses and in Mendelian Randomization. We find that a higher salary is associated with a younger age of ALS onset taking into account sex, occupation, years of education, and clinical presentation.

## Introduction

Amyotrophic lateral sclerosis (ALS) is a genetically complex neurodegenerative disease of mid-life, which results in the progressive loss of motor neurons that control voluntary muscles, with death occurring on average two years from the first symptoms (1). Socioeconomic status is defined as a measure of one’s shared economic and social status and tends to be positively associated with better health (2). Since environmental exposure is considered a modifiable risk factor for ALS, and socioeconomic status and environmental exposure are strongly correlated, socioeconomic factors are likely to contribute to ALS risk. We therefore sought to test the hypothesis that socioeconomic factors are risk factors for ALS.

There are three common measures of socioeconomic status: education, income, and occupation (3). Socioeconomic status has an established effect on the incidence and prevalence of many diseases. For example, socioeconomic status is associated with increased cardiovascular disease risk in men and women (4) as well as with many types of cancer, and respiratory diseases (5). Moreover, Mendelian Randomization has demonstrated a relationship between better educational attainment as a socioeconomic factor and a lower risk of Alzheimer’s disease (6).

One study has suggested that greater educational attainment is associated with increased ALS risk (7). This observation might indicate a relationship between socioeconomic status and ALS risk. Similarly, income has been explored as an ALS risk factor, but only as a proxy for other factors, not because it corresponds to socioeconomic status (8).

Occupational exposures have also been studied as potential ALS risk factors but in isolation as environmental risk factors rather than as indicators of socioeconomic status (9). For example, military service has been found to increase the risk of ALS (10) but this is assumed to be a result of environmental exposure resulting from deployment to a conflict zone rather than from any interaction with socioeconomic status.

Since socioeconomic status is an established criterion influencing disease risk, we set out to explore the relationship between socioeconomic status, age of onset of ALS, and risk of ALS. Because there is a genetic component to socioeconomic status, it is possible to leverage such data to explore the relationship between socioeconomic status and ALS indirectly. We further sought to validate our findings using convergent evidence from genetic studies.

## Methods

### Participants

Data were obtained from the Motor Neurone Disease Association of England, Wales, and Northern Ireland (MNDA) Collections as part of the MNDA Epidemiology Study, REC reference 07/MRE01/57 as previously described (11). In brief, people who had an El Escorial diagnosis of definite, probable, possible, or suspected ALS between 2008 and 2013 were surveyed. To ensure a representative incident population rather than a prevalence cohort that would likely be skewed by survival effects, people with ALS were recruited at secondary centers like district general hospitals. These centers served as the data collection hubs for three tertiary centres in London, Sheffield, and Birmingham. General practitioners from the general practice of the person with ALS were asked to invite 10 healthy controls from the same area to participate in the study via post, expecting that on average, one would agree. In general, each practice contributed a single control participant. The research team matched people on age within 5 years of the person with ALS and on gender in a 1:1 ratio. Consenting participants took part in a telephone interview that included socioeconomic status questions.

Dataset validity was assessed by analysis of demographic and phenotypic variables for which typical ALS population values are known. These were the male-female ratio, distribution of bulbar and limb onset of first symptoms, sex distribution of bulbar onset disease, and age of onset.

### Determination of socioeconomic category

We used three determinants of socioeconomic status: years of education, mean salary over the duration of active employment, and occupation.

Years of education included the entire educational history provided. Income was categorized in bands of £10,000 per year, with those earning £70,000 per year or more grouped together. Occupation was classified using two different algorithms for sensitivity testing, both using the National Statistics Socio-economic Classification. The first algorithm derived the Occupational Code and the second derived an 8-category analytic coding (ACNES) (12).

### Statistical analysis

IBM SPSS v29.0 was used for statistical testing throughout.

Logistic regression was used to test association, with ALS risk as the dependent variable, and site of disease onset, sex, income, occupation, and years of education as independent covariates. Linear regression with age of onset as the dependent variable was also used, again using the same covariates (except the age of onset). Analyses were done as univariate tests followed by a multivariate model. Non-ordinal categorical variables were coded as dummy variables to allow analysis.

### Mendelian randomization analysis

To search for convergent evidence supporting our results, we used genetic data from people with ALS and controls in a Mendelian randomization (MR) framework to investigate a potential causal relationship between salary, education, and occupation with the ALS risk using MR Base http://www.mrbase.org. Mendelian randomization minimizes confounding and information bias, which is frequently present in traditional epidemiological research. We conducted a two-sample Mendelian randomization analysis employing inverse-variance weighted, weighted median, and MR Egger regression analyses. We accessed genome-wide association studies of years of education from the UK Biobank (*n* = 293,723, European population), income (*n* = 4982, European population), and a meta-analysis of ALS risk (*n* = 12,577) as the outcome in each case. These studies were used as convenience samples since large population-based datasets are required. While there may be between-country differences, we could not easily account for those given the lack of multiple alternative data collections. There was no association study suitable for testing occupation or occupational category as an exposure.

## Results

There were 413 participants who provided informed consent, and 405 of these undertook a telephone interview about their lifestyle. Of those, 284 were cases, and 121 were controls. There were 226 men and 179 women. The mean age of onset was 61.2 years. There were 76 people with bulbar onset ALS and 193 who had limb onset disease; eight presented with respiratory onset and seven patients with mixed onset.

### Regression analysis results

Logistic regression showed no association between any socioeconomic variable and ALS risk.

Univariate linear regression showed no association between income category and age of ALS onset (beta = −0.071, *p* = 0.16). Univariate analysis of years of education and occupational category did not show an association with ALS age of onset.

Because socioeconomic status is a complex construct, being comprised of multiple factors (here primarily, years of salary, years of education, and occupation), we used a multivariate model to investigate these factors simultaneously as predictors of risk and age of onset, incorporating other potential confounding variables: gender and site of onset.

Multivariate logistic regression showed no association between any socioeconomic variable and ALS risk (Table 1).

**Table 1.** Results of multivariate logistic regression.

	Beta	*SE*	*p*-Value
Years of education	0.005	0.02	0.82
Being male	−0.023	0.22	0.92
Occupational code	N/A	N/A	0.95
Salary	−0.002	0.01	0.78

There is no association between ALS risk and socioeconomic variables.

Multivariate linear regression showed an association of salary with ALS using the Occupational Code as the occupation classifier (beta = −0.157, *p* = 0.011, adjusted *r*^2^ = 0.04; Table 2). The negative slope (beta) shows that a higher salary is associated with a younger age of onset. Repeating the analysis with ACNES as the occupation classifier confirmed the result (beta = −0.106, *p* = 0.05, adjusted *r*^2^ = 0.06; Table 3).

**Table 2.** Results of multivariate linear regression.

	Beta	*SE*	*p*-Value
Being male	−2.1	1.73	0.23
Bulbar onset	2.96	2.04	0.15
Salary	−0.157	0.06	0.011
Occupation code	N/A	N/A	0.11
Years of education	0.05	0.15	0.74

Salary is significantly associated with ALS age of onset even after accounting for other variables, such as the site of onset and gender.

**Table 3.** Results of multivariate linear regression sensitivity analysis.

	Beta	*SE*	*p*-Value
Being male	−2.7	1.7	0.12
Bulbar onset	1.74	2.0	0.39
Salary	−0.106	0.053	0.05
Occupation code	N/A	N/A	0.06
Years of education	−0.07	0.16	0.64

Salary remains associated with ALS age of onset when using a different occupation classifier algorithm.

### Mendelian randomization results

**Figure 1.**
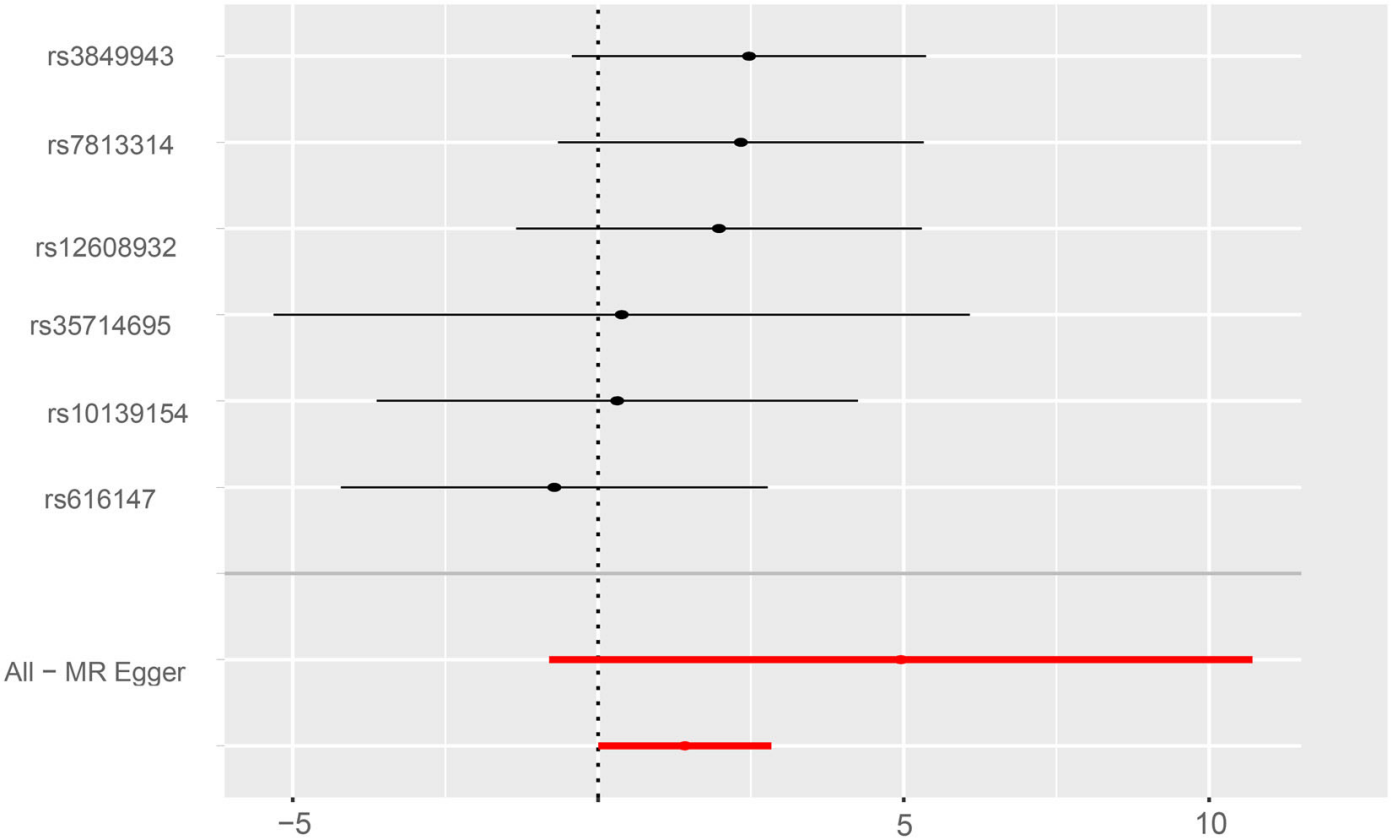
Estimate of causal relationship between average household income before tax and risk of amyotrophic lateral sclerosis.

Mendelian randomization analysis provided support for our findings, with Inverse variance weighted and weighted median results showing an association between ALS and salary (Table 4, Figure 1). We would hypothesize that the association between income and ALS is a result of unmeasured variables rather than a direct causal relationship. Consistent with this hypothesis, the MR Egger results suggest that the association is a result of horizontal pleiotropy rather than direct causation.

**Table 4.** Inverse variance weighted and weighted median analysis showed a significant association between household income and ALS risk.

	Number of SNPs	Beta	*SE*	*p*-Value
Inverse variance weighted	6	1.42	0.723	0.050
Weighted median	6	2.06	0.899	0.022
Weighted mode	6	2.24	1.178	0.116
MR Egger	6	4.95	2.937	0.167

## Discussion

We found that a higher income category is independently associated with a younger age of onset of ALS but not with a higher risk compared with controls. The finding that income is related to age of ALS onset is unexpected, and we therefore attempted to corroborate our findings using Mendelian randomization. Typically, the Inverse Variance Weighted method is interpreted as the overall result, and in our case, it is exactly at the threshold for significance, with *p* = 0.05. The Weighted Median method is robust to poor instrumental variables that might otherwise affect the Inverse Variance Weighted method and supports a causal relationship between income and ALS risk, but the lack of a confirmed association using MR Egger suggests that horizontal pleiotropy may be the explanation, and that the apparently causal relationship is therefore spurious. Genetic variants do not directly determine income but have an indirect effect through their associations with traits like educational attainment, cognitive ability, personality, and health conditions. These traits can then impact socioeconomic status and income through various pathways. However, each genetic variant has a very small effect, and environmental and social factors play a much larger role in income variation across individuals and populations. Horizontal pleiotropy occurs when the variant has an effect on disease outside of its effect on the exposure in Mendelian randomization. In other words, the genetic variants that influence income also influence other factors that increase ALS risk. Nevertheless, the Mendelian randomization analysis provides convergent evidence for our finding since it shows an association, and it is unlikely that salary is a direct cause of younger onset of ALS, so the finding of possible horizontal pleiotropy is consistent with expectations.

It might be expected that occupation influences the likelihood of ALS because occupation is a proxy for other risk factors. For example, an association has been previously reported between ALS risk and professional football. This has been variously attributed to the possibility of head injury, aerosol injection of pesticides during ball impact, and a predisposition to ALS in individuals engaging in sport because of a genetic component (13). The causation of ALS may be the result of a multistep cascade (14,15). Occupational exposures might therefore be expected to impact ALS risk. Our finding of no association is therefore surprising but maybe because of the difficulty in classifying occupations in a meaningful way for such an analysis given that the mechanism of non-genetic (i.e. apparently sporadic) ALS is unknown. Without a clear understanding of how environmental or occupational factors contribute to ALS pathogenesis, it becomes difficult to group occupations in a way that accurately captures relevant exposures or risk factors. Our relatively small sample size also means that any heterogeneity in the classification of occupations will significantly reduce already reduced power. We have tried to overcome this problem by using two different algorithms for occupational classification.

A great deal of previous research has investigated the impact of income on disease risk factors but on a population scale, for example, blood pressure risk for cardiac diseases has been examined in high- and low-income countries (16–19). The impact of salary on the risk of diseases is the subject of fewer investigations but also includes examining the effect of income on disease progression or survival. In the case of kidney diseases, for instance, there is a consistent and positive correlation between higher income percentile and longer life expectancy (20).

Consistent with our findings on a small population level, a previous analysis examing the Global Burden of Diseases, Injuries and Risk Factors Study 2016 across 21 world regions (21) found that in general, the incidence and prevalence of ALS is higher in countries with a higher sociodemographic index. Explanations considered included improved awareness, shorter time to diagnosis, and increased access to multidisciplinary care. Our findings are consistent with these reports since we show that a higher income is associated with a younger onset of ALS, not protection. This is seen sometimes in other conditions. For example, higher incomes are associated with an increased risk of anorexia nervosa (22). Similarly, Down syndrome, which is associated with older age at conception in mothers, would also be expected to correlate with the higher income of an older person. Given that education is linked to a mother’s later age at conception, it is plausible that the relationship between income and the likelihood of Down syndrome is due to the impact of education on salary (23).

A limitation of this study is that the lifetime income would be expected to rise with increasing age, and therefore there is confounding between the predictor variable, income, and the dependent variable, age of ALS onset. However, this observation would be expected to result in an association of higher income with increasing age of onset, not what we found, and our analysis is therefore conservative concerning our findings. Also, the imbalanced nature of 284 cases *vs.* 121 controls means that bias is more likely.

We have found that a higher mean lifetime salary is associated with a younger age of onset of ALS. To fully understand this link and the underlying causal mechanisms, more research is required.

## Data Availability

The data presented in this study are available upon request.

## References

[1] Brown RH, Al-Chalabi A. Amyotrophic lateral sclerosis. N Engl J Med. 2017;377:162–172. 10.1056/NEJMra1603471 28700839

[2] Oh J, An JW, Oh SI, Oh KW, Kim JA, Lee JS, et al. Socioeconomic costs of amyotrophic lateral sclerosis according to staging system. Amyotroph Lateral Scler Frontotemporal Degener. 2015;16:202–8. 25646865 10.3109/21678421.2014.999791

[3] Navarro-Carrillo G, Alonso-Ferres M, Moya M, Valor-Segura I. Socioeconomic status and psychological well-being: revisiting the role of subjective socioeconomic status. Front Psychol. 2020;11:1303. 32587560 10.3389/fpsyg.2020.01303 PMC7298147

[4] Hergault H, Hauguel-Moreau M, Pépin M, Beauchet A, Josseran L, Rodon C, et al. Impact of neighbourhood socio-economic status on cardiovascular risk factors in a French urban population. Eur J Prev Cardiol. 2022;29:2142–4. 35894217 10.1093/eurjpc/zwac155

[5] Kawakatsu Y, Koyanagi YN, Oze I, Kasugai Y, Morioka H, Yamaguchi R, et al. Association between socioeconomic status and digestive tract cancers: a case-control study. Cancers. 2020;12:3258. 33158224 10.3390/cancers12113258 PMC7694284

[6] Larsson SC, Traylor M, Malik R, Dichgans M, Burgess S, Markus HS, CoSTREAM Consortium, on behalf of the International Genomics of Alzheimer’s Project. Modifiable pathways in Alzheimer’s disease: Mendelian randomization analysis. BMJ. 2017;359:j5375. 29212772 10.1136/bmj.j5375 PMC5717765

[7] Zhang L, Tang L, Xia K, Huang T, Fan D. Education, intelligence, and amyotrophic lateral sclerosis: a Mendelian randomization study. Ann Clin Transl Neurol. 2020;7:1642–7. 32810387 10.1002/acn3.51156 PMC7480912

[8] Beard JD, Kamel F. Military service, deployments, and exposures in relation to amyotrophic lateral sclerosis etiology and survival. Epidemiol Rev. 2015;37:55–70. 25365170 10.1093/epirev/mxu001 PMC4325667

[9] Swash M, Eisen A. Hypothesis: amyotrophic lateral sclerosis and environmental pollutants. Muscle Nerve. 2020;62:187–91. 32134532 10.1002/mus.26855

[10] Roberts AL, Johnson NJ, Chen JT, Cudkowicz ME, Weisskopf MG. Race/ethnicity, socioeconomic status, and ALS mortality in the United States. Neurology 2016;87:2300–8. 27742817 10.1212/WNL.0000000000003298 PMC5135021

[11] Opie-Martin S, Jones A, Iacoangeli A, Al-Khleifat A, Oumar M, Shaw PJ, et al. UK case control study of smoking and risk of amyotrophic lateral sclerosis. Amyotroph Lateral Scler Frontotemporal Degener. 2020;21:222–7. 32301340 10.1080/21678421.2019.1706580 PMC7261396

[12] The National Statistics Socio-Economic Classification (NS-SEC). 2021.

[13] Daneshvar DH, Mez J, Alosco ML, Baucom ZH, Mahar I, Baugh CM, et al. Incidence of and mortality from amyotrophic lateral sclerosis in National Football League Athletes. JAMA Netw Open. 2021;4:e2138801. 34910152 10.1001/jamanetworkopen.2021.38801 PMC8674746

[14] Hoffman HI, Bradley WG, Chen CY, Pioro EP, Stommel EW, Andrew AS. Amyotrophic lateral sclerosis risk, family income, and fish consumption estimates of mercury and omega-3 PUFAs in the United States. Int J Environ Res Public Health. 2021;18:4528. 33923256 10.3390/ijerph18094528 PMC8123167

[15] Al-Chalabi A, Hardiman O. The epidemiology of ALS: a conspiracy of genes, environment and time. Nat Rev Neurol. 2013;9:617–28. 24126629 10.1038/nrneurol.2013.203

[16] Mills KT, Stefanescu A, He J. The global epidemiology of hypertension. Nat Rev Nephrol. 2020;16:223–37. 32024986 10.1038/s41581-019-0244-2 PMC7998524

[17] Braveman PA, Cubbin C, Egerter S, Chideya S, Marchi KS, Metzler M, et al. Socioeconomic status in health research: one size does not fit all. JAMA 2005;294:2879–88. 16352796 10.1001/jama.294.22.2879

[18] Krumholz HM, Bernheim SM. The role of socioeconomic status in hospital outcomes measures. Ann Intern Med. 2015;162:670. 10.7326/L15-5087-2 25939004

[19] Kong Y, Shaver LG, Shi F, Mu H, Bu W, Etchegary H, et al. The effects of cancer beliefs and sociodemographic factors on colorectal cancer screening behaviours in Newfoundland and Labrador. Healthcare. 2022;10:2574. 36554096 10.3390/healthcare10122574 PMC9778754

[20] Lang J, Shlipak MG. Kidney disease, income, and life expectancy. Am J Kidney Dis. 2016;68:674–6. 27772630 10.1053/j.ajkd.2016.07.004

[21] Disease and Injury Incidence and Prevalence Collaborators. Global, regional, and national incidence, prevalence, and years lived with disability for 328 diseases and injuries for 195 countries, 1990-2016: a systematic analysis for the Global Burden of Disease Study 2016. Lancet. 2017;390:1211–59. 28919117 10.1016/S0140-6736(17)32154-2 PMC5605509

[22] Sonneville KR, Lipson SK. Disparities in eating disorder diagnosis and treatment according to weight status, race/ethnicity, socioeconomic background, and sex among college students. Int J Eat Disord. 2018;51:518–26. 29500865 10.1002/eat.22846

[23] Dzurova D, Pikhart H. Down syndrome, paternal age and education: comparison of California and the Czech Republic. BMC Public Health. 2005;5:69. 15963229 10.1186/1471-2458-5-69 PMC1166564

